# Modest induction of phase 2 enzyme activity in the F-344 rat prostate

**DOI:** 10.1186/1471-2407-6-62

**Published:** 2006-03-15

**Authors:** Sunita B Jones, James D Brooks

**Affiliations:** 1 Department of Urology, Stanford University School of Medicine, Stanford, California 94305, USA

## Abstract

**Background:**

Prostate cancer is the most commonly diagnosed malignancy in men and is thought to arise as a result of endogenous oxidative stress in the face of compromised carcinogen defenses. We tested whether carcinogen defense (phase 2) enzymes could be induced in the prostate tissues of rats after oral feeding of candidate phase 2 enzyme inducing compounds.

**Methods:**

Male F344 rats were gavage fed sulforaphane, β-naphthoflavone, curcumin, dimethyl fumarate or vehicle control over five days, and on the sixth day, prostate, liver, kidney and bladder tissues were harvested. Cytosolic enzyme activities of nicotinamide quinone oxidoreductase (NQO1), total glutathione transferase (using DCNB) and mu-class glutathione transferase (using CDNB) were determined in the treated and control animals and compared.

**Results:**

In prostatic tissues, sulforaphane produced modest but significant increases in the enzymatic activities of NQO1, total GST and GST-mu compared to control animals. β-naphthoflavone significantly increased NQO1 and GST-mu activities and curcumin increased total GST and GST-mu enzymatic activities. Dimethyl fumarate did not significantly increase prostatic phase 2 enzyme activity. Compared to control animals, sulforaphane also significantly induced NQO1 or total GST enzyme activity in the liver, kidney and, most significantly, in the bladder tissues. All compounds were well tolerated over the course of the gavage feedings.

**Conclusion:**

Orally administered compounds will induce modestly phase 2 enzyme activity in the prostate although the significance of this degree of induction is unknown. The 4 different compounds also altered phase 2 enzyme activity to different degrees in different tissue types. Orally administered sulforaphane potently induces phase 2 enzymes in bladder tissues and should be investigated as a bladder cancer preventive agent.

## Background

The most commonly diagnosed cancer among men, prostate cancer will account for nearly 30,000 deaths in the United States in 2005 and cause countless men to suffer significant morbidity [1]. Accumulating evidence implicates oxidative damage, possibly due to prostatic inflammation, as an important contributor to prostate carcinogenesis [2]. Some human prostate cells appear to acquire increased susceptibility to oxidative DNA damage because they lack expression of glutathione S-transferase-π (GSTP1) due to somatically acquired methylation of deoxycytidine residues in "CpG islands" in the 5'-regulatory region of the GSTP1 gene early in prostate carcinogenesis [3-6]. GSTP1 is an important member of the class of enzymes (phase 2 enzymes) that protect cells against electrophilic compounds, including many carcinogens and oxidative species [7]. Strategies to induce the expression and activity of phase 2 enzymes have been shown to protect against carcinogenesis in a variety of organ sites and across several species [8,9]. Since prostate cancer appears to be uniquely deficient in the phase 2 enzyme GSTP1, a rational prevention strategy might be to compensate for GSTP1 loss by global induction of phase 2 enzymes *within *the prostate.

A number of compounds effective at inducing phase 2 enzyme activity have been identified by screening for nicotinomide quinone oxidoreductase (NQO1) enzymatic induction in the Hepa 1c1c7 cell line [10-12]. Compounds effective at inducing phase 2 enzymatic activity in Hepa 1c1c7 cells *in vitro *have been found to be effective at inducing the phase 2 enzyme response *in vivo*, and several of these compounds have also been demonstrated to prevent against carcinogen induced tumors in animal models [11,13]. However, compounds that induce NQO1 activity in liver-derived Hepa 1c1c7 cells do not always produce induction in liver cells *in vivo*, and can vary in their effectiveness at inducing phase 2 enzymes in different tissues [14-16]. For instance, both *tert*-butyl-4-hydroxyanisole (BHA) and dimethyl fumarate are effective at inducing NQO1 activity in Hepa 1c1c7 cells *in vitro*, but in CD-1 mice, only BHA induces NQO1 activity in the liver (6-fold), in addition to the lung and kidney (2-fold), but not in the stomach and colon [17]. Dimethyl fumarate, on the other hand, induces NQO1 enzymatic activity in the forestomach, small intestine, kidneys and lungs, but produces little change in NQO1 activity in the liver [11].

To identify compounds effective at inducing phase 2 enzymes in human prostate cells, we have carried out a comprehensive screen of candidate phase 2 enzyme inducing agents in human prostate cancer cells *in vitro *and identified compounds from several chemical classes that were effective at producing modest increases in NQO1 enzymatic activity [18]. Notably, the pattern of NQO1 induction across compounds differed between prostate cancer cell lines and a human liver cell line, suggesting that there could be significant differences in the response of prostate cells to phase 2 enzyme inducing agents compared to other tissue types. We also have demonstrated that sulforaphane, an isothiocyanate found in cruciferous vegetables, induces NQO1, glutathione synthetic enzymes and glutathione transferases in several human prostate cancer cells [19].

Although we have identified compounds effective at inducing phase 2 enzymes in prostate cells *in vitro*, the possibility of inducing phase 2 enzyme response in the prostate *in vivo *has not been tested. We selected 4 candidate phase 2 enzyme inducing agents effective in prostate cells *in vitro *and tested whether they could induce phase 2 enzyme enzymatic activity in the prostates of F344 rats *in vivo*. After 5 days of gavage feeding with each of candidate compounds or vehicle alone, global GST activity, isozyme GST-mu and NQO1 activity were assessed in the prostate, liver, kidney and bladder tissues of male rats.

## Methods

### Materials

Purified sulforaphane was obtained from LKT labs (St. Paul, MN), curcumin, β-naphthoflavone (BNF), propylene glycol, dimethyl fumarate (DMF), 1,2-dichloro-4-nitrobenzene (DCNB) and 1-chloro-2,4-dinitrobenzene (CDNB) from Sigma-Aldrich Inc. (St. Louis, MO), and AIN 76A diet from Research Diets (New Brunswick, NJ). Male F-344 rats were purchased from Jackson labs (Bar Harbor, ME).

### Treatment of animals and tissue collection

Eight-week-old male F344 rats were housed in microisolator cages in groups of 2 or 3 animals per cage. Mean body weight of the rats was 188 ± 4.0 g at the start of the study, and animals had access to AIN 76A diet and water *ad libitum *over the duration of the study. Animals were randomly divided into 5 groups of 10 animals corresponding to each of the 4 test compounds and a control group that received vehicle alone. All compounds were mixed fresh each day by either dissolving or suspending them in 100 μl propylene glycol at the following doses: sulforaphane 50 mg/Kg/day [13], curcumin 45 mg/Kg/day [20], β-naphthoflavone 41 mg/Kg/day [15] and dimethyl fumarate 37.5 mg/Kg/day [11]. Doses were chosen that had been reported to be non-toxic and effective at inducing phase 2 enzymes in other model systems. Compounds or propylene glycol were administered in a single dose once a day by gavage at doses corrected for the body weight of each animal. The gavage feeding was carried out after the rats received isofluorane inhalation anesthesia and involved minimal trauma. Animals recovered rapidly from this agent and were observed until fully awake in their cages. All rats were monitored for infection or toxicity to prevent suffering, and there were no obvious signs of discomfort, distress, or pain over the duration of the study. One animal in the sulforaphane group died shortly after the first gavage feeding, and another died after the second feeding, both apparently due to aspiration of the dose. Necropsy did not reveal any gross abnormalities of any organs. On the morning of the sixth day, the rats were sacrificed by CO_2 _asphyxiation approximately 24 hours after the last dose.

The rats were housed at the Animal Care Facility at the Stanford University School of Medicine in compliance with PHS Policies on Humane Care and Use of Laboratory Animals. All work was carried out under Administrative Panel on Laboratory Animal Care approved protocols. All animals were under strict veterinarian care of the Department of Comparative Medicine in compliance with all Federal and State regulations to assure proper and humane treatment.

### Collection of tissues and preparation of cytosol

The liver, kidneys, bladder and the prostate tissues were removed, weighed and snap frozen in liquid Nitrogen, and stored in -80°C until processed for the enzyme assays. Cytosols were prepared from the harvested tissues by homogenization in 0.25 M sucrose and centrifugation at 5000 × g for 20 minutes at -4°C. 0.2 volume of 0.1 M CaCl_2 _in 0.25 M sucrose was added to the supernatant and, after incubation on ice for 30 minutes, samples are centrifuged at 15,000 × g at -4°C for 20 minutes.

### Enzyme assays

Total glutathione transferase enzyme activity was determined using 1,2-dichloro-4-nitrobenzene (DCNB) and GST mu activity was measured using 1-chloro-2,4-dinitrobenzene (CDNB) according to the procedure of Habig et al. [21]. Cytosols (50 μl) were added to 150 μl 0.1 M phosphate buffer pH 6.5 buffer with 1 mM GSH, 1 mM CDNB or DCNB, and 1% BSA, mixed and optical absorbance was read at 340 nm at 30 sec intervals over 5 minutes. Because of high specific activity, liver samples were diluted 5-fold. GST mu activity was not measured in the bladder samples. Quinone reductase activity was determined by the rate of the NADPH-dependent, menadione-coupled reduction of 3-(4,5-dimethylthiazol-2-yl)-2,5-diphenyltetrazolium bromide in 96-well microtire plates as described previously [18,19,22]. Enzyme activities were normalized to total cytosolic protein measured according to the Bradford method [23]. All assays and protein measurements were performed in triplicate. Mean enzyme specific activities for tissues from animals in each group were calculated and the fold-induction enzyme specific activities determined by taking a ratio of log-transformed inducer-treated enzyme activities to the controls. The 95% confidence intervals for the fold-induction of enzyme specific activities of the inducer-treated animals compared to controls were calculated on log-transformed data and the results back-transformed to the fold-scale.

## Results

All compounds were well tolerated by the animals and there was no apparent toxicity over the duration of the study. None of the compounds affected the relative weights of the prostate, kidneys, liver or bladder after gavage feedings over 5 days. Body weights did not differ significantly between the groups fed candidate compounds and the control animals (Table 1). However, compared to initial body weights, there was an 8% decrease in body weight in the sulforaphane treated group after 5 days (*P *= 0.03), and non-significant increases (1–5%) in the body weights of the other 4 groups.

**Figure 1:**
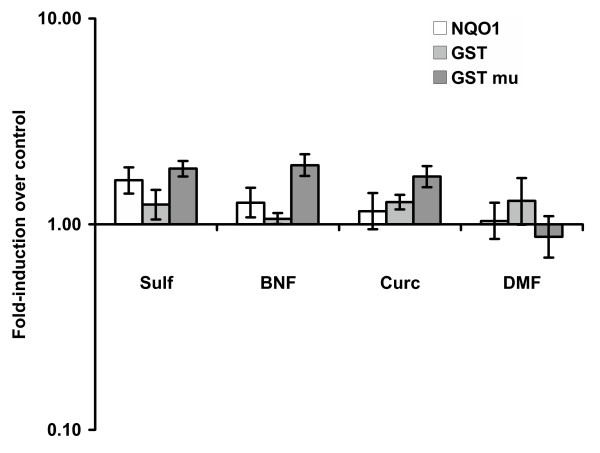
Fold-induction of phase 2 enzymes NQO1, total GST and GST-mu activity in the prostate tissue of male F344 rats treated for 5 days with 50 mg/Kg Sulforaphane (n = 8), 45 mg/Kg Curcumin (n = 10), 41 mg/Kg β-naphthoflavone (n = 10) and 37.5 mg/Kg Dimethylfumarate (n = 10). Fold-induction is the ratio of specific activity of treated against control animals (n = 10) and is plotted on a log scale. Data represent average of assays in triplicate. Data are mean and 95% confidence intervals of animals in each group.

Sulforaphane treated animals showed significantly higher NQO1, total GST and GST-mu enzymatic activities in their prostate tissues compared to control animals (Figure 1 and Tables 2, 3 and 4) although the degree if increase was modest (1.2- to 1.8-fold). Compared to controls, β-naphthoflavone treated animals showed small, statistically significant higher levels of NQO1 activity, no differences in total GST enzymatic activity, and moderately elevated GST-mu activity in the prostate. Curcumin treated animals also displayed significantly higher total GST and GST-mu activities in prostate tissues over control levels, although, again, the differences were modest. Prostate tissues from dimethyl fumarate treated animals did not show differences in NQO1, total GST or GST-mu enzymatic activities compared to controls.

**Figure 2:**
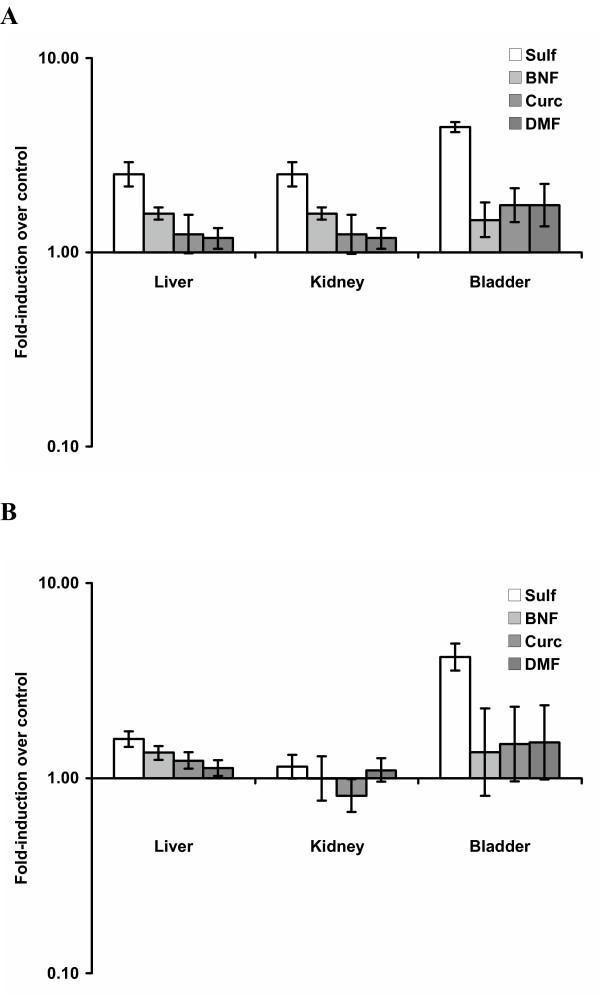
Fold-induction in NQO1 (A) and total GST (B) enzyme activities in the liver, kidney and bladder tissues of male F344 rats treated for 5 days with 50 mg/Kg Sulforaphane (n = 8), 45 mg/Kg Curcumin (n = 10), 41 mg/Kg β-naphthoflavone (n = 10) and 37.5 mg/Kg Dimethylfumarate (n = 10). Fold-induction is the ratio of specific activity of treated against control animals (n = 10) and is plotted on a log scale. Data represent the average of assays in triplicate. Data are mean 95% confidence intervals of animals in each group.

The effects of sulforaphane, β-naphthoflavone, curcumin and dimethyl fumarate on phase 2 enzyme activity in the liver, kidney and bladder in many ways paralleled that observed in the prostate. Liver tissues from animals treated with sulforaphane, β-naphthoflavone, and to a lesser extent dimethyl fumarate, showed modestly higher NQO1 enzyme activity compared control animals, while curcumin appeared to have no effect (Figure 2A and Table 2). All four compounds resulted in significantly higher total glutathione transferase enzymatic activity in the livers of treated animals compared to controls, and sulforaphane produced the greatest elevation (Figure 2B and Table 3). Somewhat paradoxically GST-mu activity levels in the liver did not differ significantly between animals treated with inducer compounds and controls and were actually lower in sulforaphane-treated animals (Table 4). NQO1 enzymatic activity was also higher in the kidney tissues of the sulforaphane, β-naphthoflavone and dimethyl fumarate treated animals compared to controls, while NQO1 enzyme activities in curcumin treated animals matched those seen in controls (Figure 2A and Table 2). On the other hand, kidney levels of total GST and GST-mu enzymatic activity were no different between the 4 inducer compound treated groups and the controls except for induction of GST-mu by curcumin (Figure 2B and Tables 3 and 4). Interestingly, NQO1 and total glutathione transferase enzymatic activities were dramatically higher in the bladder tissues of the sulforaphane treated animals compared to the controls (4.4-fold and 4.2-fold, respectively) (Figure 2A and 2B, and Tables 2 and 3). NQO1 enzyme activities in bladder tissues were also significantly increased over controls in the animals treated with β-naphthoflavone, curcumin and dimethyl fumarate, although the differences were not as marked as in the sulforaphane-treated animals. Total GST enzyme specific activities did not differ significantly from control bladder tissues for any of the three compounds.

## Discussion

We have previously identified compounds effective at producing modest increases in NQO1 enzymatic activity in human prostate cells *in vitro *[18,19] and selected 4 compounds for testing whether they could produce induction of phase 2 enzyme activity *in vivo*. We selected sulforaphane, dimethyl fumarate and cucumin since they were among the most potent NQO1 inducing agents in prostate cells *in vitro*, have been reported to be monofunctional inducers (i.e. induce phase 2 enzymes primarily), and have been administered to animals without toxicity previously [11,13,20]. β-naphthoflavone, a bifunctional (phase 1 and 2) enzyme inducing compound, was selected because of its documented ability to induce phase 2 enzyme activity in rodent tissues *in vivo *and for comparison to the other compounds since it increased NQO1 activity to a lesser degree in the prostate cells *in vitro *[15].

We have demonstrated that orally administered agents can produce modest increases in phase 2 enzyme activity in prostate tissues *in vivo*. We have shown previously that sulforaphane, curcumin, dimethyl fumarate and, to a lesser degree, β-naphthoflavone will induce modest increases NQO1 enzymatic activity in prostate cancer cells *in vitro *[18,19]. Effective induction *in vivo *depends on candidate phase 2 enzyme inducing compounds being absorbed from the gastrointestinal tract, and those compounds or their active metabolites reaching the prostate, being absorbed from the circulation and acting in prostate cells in the context of their physiological environment. Our finding of even modest induction of phase 2 enzyme activity implies that each of these pharmacokinetic constraints can be overcome, and suggests that phase 2 enzyme induction by orally administered agents could represent a possible prostate cancer prevention strategy. However, whether the modest increases in phase 2 enzyme activity induced by sulforaphane, dimethyl fumarate, cucumin and β-naphthoflavone are sufficient to prevent prostate cancer is unknown and remains to be tested.

The F344 rat will develop prostate adenocarcinoma after chronic administration of 2-amino-1-methyl-6-phenylimidazo [4,5-b]pyridine (PhIP) and is one of the few carcinogen-induced animal models of prostate cancer [24]. We selected this strain of rats to test to the possibility of phase 2 enzyme induction in the prostate as a prelude to future experiments designed to test whether phase 2 enzyme induction in the prostate could prevent PhIP-induced prostate cancers. The degree of increase of NQO1, total GST and GST-mu enzymatic activities in the prostate tissues we observed was modest, and lower than that reported in other model systems where phase 2 enzyme inducing compounds have been documented to prevent carcinogenesis [25]. However, in man, prostate cancer develops over decades, raising the possibility that chronic, low-level phase 2 enzyme induction might be sufficient to prevent the disease. Furthermore, modest induction of phase 2 enzymes (NQO1 and total GST), virtually identical to those reported in the present study, have been observed in the liver tissues of F344 rats treated with sulforaphane and sulforaphane nitrile derived from cruciferous vegetables [26]. Cruciferous vegetables will decrease the incidence preneoplastic lesions in the colon and liver when fed simultaneously with the carcinogen 2-amino-3methylimidazo [4,5-*f*]quinoline (IQ) to F344 rats [27]. Therefore, even relatively modest induction of phase 2 enzymatic activity can be sufficient to protect against carcinogenesis. Whether similar protection against prostatic carcinoma will occur requires further testing. The finding that consumption of cruciferous vegetables has been associated with a decreased risk of subsequent prostate cancer diagnosis, coupled with the ability of orally administered sulforaphane to induce phase 2 enzyme activity in the prostate, suggests that phase 2 enzyme induction within the prostate is a potential prostate cancer preventive strategy and sulforaphane is a candidate preventive agent [28,29].

In agreement with previous observations, we found that each compound showed differing efficacy at inducing phase 2 enzyme activity in different tissue types. The kidney, for instance, showed little induction of the glutathione transferases, while the GSTs were readily induced in the liver, bladder and prostate. Prochaska et al. have reported that the induction patterns of derivatives of tert-butyl-4-hydroxyanisole (BHA) varied in their efficacy of phase 2 enzyme induction, differed in the spectrum enzymes they each induced and differed in their effectiveness between the liver, esophagus, forestomach, colon, kidney and lung [17]. Similarly, Spencer et al. found that in CD-1 mice, dimethyl fumarate induced NQO1 enzymatic activity in the forestomach, small intestine, kidneys and lungs, but failed to induce NQO1 activity in the liver, similar to our findings in the F344 rat [11]. They also found that the patterns of induction of total GST, GST-mu, and NQO1 enzymatic activities differed between compounds and by tissue type. Van Lieshout et al. have also described differences in phase 2 enzyme responsiveness in the tissues of Wistar rats after treatment oltipraz, α-tocopherol, β-carotene, and phenethyl isothiocyanate [16].

The reasons for the differences in the responsiveness of phase 2 enzymes between tissues are currently unknown, but likely are a reflection of tissue-specific expression of transcriptional regulators or enzyme cofactors. The difference in responsiveness between tissues does have important implications in the design and interpretation of preventive intervention trials involving phase 2 enzyme induction. For instance, cancers that arise from the oral ingestion of carcinogens, such as the 9,10-dimethyl-1,2-benzanthracene rat model of breast cancer or aflatoxin-induced hepatocellular carcinomas in man, might best be prevented by oral ingestion of agents that will induce phase 2 enzymes and inactivate these carcinogens in the gut and liver [13,30,31]. However, accumulating evidence suggests that for prostate cancer induction of phase 2 enzymes *within *the prostate might best protect against carcinogenesis.

No environmental carcinogens have been identified as causing human prostate cancer. Accumulating evidence implicates endogenous oxidative damage as one important contributor to prostate carcinogenesis [2]. Prostate cancer increases with age and may be related to inflammatory conditions of the prostate such as prostatitis [32]. Androgens are a known requisite to prostate cancer development. Ripple et al. have demonstrated that treatment of the prostate cancer cell line LNCaP with androgens produces a burst of oxidative stress in these cells with generation of reactive oxygen species, increased lipid peroxidation and a depletion of intracellular glutathione stores [33-35]. Furthermore, Malins et al. have described progressive alterations in DNA structure between normal, BPH and cancerous prostate tissues due to oxidative damage to the DNA template be hydroxyl free radical [36-38]. Two genes recently identified as conferring increased risk to prostate cancer in families (RNASEL and MSR2) participate in the response to infection and inflammation [39,40]. Mice engineered to not express RNASEL, for instance, are more susceptible to overwhelming bacterial infections [41]. Finally, most compounds thus far implicated as prostate cancer preventive agents act as potent antioxidants including lycopene, selenium (essential to glutathione peroxidase activity), and vitamin E [42-44].

The early and near universal loss of expression of the phase 2 enzyme GSTP1 likely renders prostate cells susceptible to local oxidative damage and transformation. GSTP1 knock-out mice treated with the polycyclic aromatic hydrocarbon 7,12-dimethylbenz anthracene and the tumor promoting agent 12-O-tetradecanoylphorbol-13-acetate show increased numbers and earlier onset of skin papillomas demonstrating that loss of expression of a single GST can contribute to carcinogenesis [45]. Since prostate cancer arises with a long latency in the context of local oxidative damage coupled with an intrinsic defect in carcinogen defenses, local induction of phase 2 enzymatic activity, even to a modest degree, could be a promising preventive strategy. Since prostate cancer develops over decades, chronic, low-level, local induction of carcinogen defenses, possibly through diet-derived agents such as sulforaphane, could represent a modest, non-toxic intervention strategy for prevention of prostate cancer, particularly for individuals at risk for the disease.

One notable finding was the significant induction of total GST and NQO1 enzymatic activities in bladder tissues of the F344 rats. Several environmental carcinogens have been linked to bladder cancer including polyaromatic hydrocarbons in tobacco smoke and aniline dyes [46]. Epidemiological studies have demonstrated that consumption of cruciferous vegetables is associated with a decreased risk of bladder cancer [47]. Sulforaphane levels peak in the serum 1–2 hours after ingestion and are cleared relatively rapidly by excretion into the urine [48]. The substantial phase 2 enzyme induction of the bladder tissues could be due to the presence of sulforaphane or its active metabolites at relatively high concentrations over prolonged time periods while they are retained in the bladder. Munday and Munday have found similar induction of NQO1 and GST activity in the bladder tissues of female Sprague-Dawley rats after oral feedings of sulforaphane and several other isothiocyanates derived from cruciferous vegetables [49]. Together, these data strongly suggest that sulforaphane and other isothiocyanates could represent promising candidate bladder cancer preventive agents.

Our study has several shortcomings. We arbitrarily selected a single daily dosing schedule based on prior studies in the literature. It is possible that other dosing schedules, perhaps different for each compound, could produce greater phase 2 enzyme induction [50]. In addition, measurement of phase 2 enzyme activity occurred 24 hours following the last dose of each compound. The serum half-life of sulforaphane is between 1–2 hours and it is possible that measurement of phase 2 enzyme activity at times less than 24 hours would reveal greater induction of enzymatic activity [48]. Finally, all animals were given isofluorane anesthesia at the time of gavage feeding, and the anesthesia could have altered phase 2 enzyme activity in the tissues. However, since both the inducer compound treated animals and controls were treated identically, the relative levels of phase 2 enzyme activity should not have been affected.

## Conclusion

We have demonstrated the possibility of inducing phase 2 enzymatic activity in the prostate tissues of F344 rats *in vivo *after oral feeding of several candidate phase 2 enzyme inducing agents. Our findings set the stage for further testing of phase 2 enzyme inducing agents in prostate cancer prevention. A first step will be to test whether phase 2 enzyme induction in the prostate will prevent prostatic cancers in animal models. If successful, additional work will be necessary to identify the phase 2 enzymes critical in cancer protection so that they can be monitored as biomarkers of effectiveness in clinical trials.

## Competing interests

The author(s) declare that they have no competing interests.

## Authors' contributions

JB designed the study. SJ and JB performed the experiments. SJ and JB analyzed the data. SJ and JB contributed to writing the paper.

## Pre-publication history

The pre-publication history for this paper can be accessed here:



## Figures and Tables

**Table 1:** Final body weight and relative organ weights of male control and inducer treated F344 rats

	**Sulforaphane**	**BNF**	**Curcumin**	**DMF**	**Control**
**Number**	8	10	10	10	10
**Final body weight (g)**	177.5 ± 10.35 *	190.3 ± 12.56	184.4 ± 13.59	190.2 ± 15.65	195.8 ± 8.52
**Liver (%)**	4.13 ± 0.22	4.84 ± 0.27	4.60 ± 0.22	4.71 ± 0.37	4.96 ± 0.22
**Kidney (%)**	0.86 ± 0.07	0.97 ± 0.04	0.91 ± 0.04	0.93 ± 0.04	0.88 ± 0.04
**Prostate (%)**	0.15 ± 0.02	0.17 ± 0.04	0.15 ± 0.03	0.15 ± 0.03	0.17 ± 0.03
**Bladder (%)**	0.03 ± 0.01	0.02 ± 0.01	0.03 ± 0.01	0.03 ± 0.01	0.02 ± 0.01

Data are mean ± SEM values. * = P < 0.05 vs. initial body weight

**Table 2:** Mean specific activity of NQO1 in nmol/min/mg of protein (Ratio of the treated/control enzyme specific activities, 95% Confidence Intervals)

	**Sulforaphane**	**BNF**	**Curcumin**	**DMF**	**Control**
**Liver**	69 (2.5, 2.2–2.9)*	43 (1.6, 1.5–1.7)*	34 (1.2, 0.99–1.6)	32 (1.2, 1.04–1.3)*	27
**Kidney**	41 (2.5, 2.2–2.9)*	26 (1.6, 1.5–1.7)*	20 (1.2, 0.99–1.6)	19 (1.2, 1.04–1.3)*	16
**Prostate**	103 (1.6, 1.4–1.9)*	80 (1.3, 1.1–1.5)*	72 (1.2, 0.95–1.4)	65 (1.04, 0.85–1.3)	63
**Bladder**	26 (4.4, 4.2–4.7)*	9.0 (1.5,1.2–1.8)*	10 (1.8, 1.4–2.1)*	10 (1.8. 1.4–2.2)*	6.0

Data are mean values of 8–10 animals in each group

* = P < 0.05 based on confidence intervals of the ratio of treated vs. control NQO1 specific activities

**Table 3:** Mean specific activity of Total GST in nmol/min/mg of protein (Ratio of the treated/control enzyme specific activities, 95% Confidence Intervals)

	**Sulforaphane**	**BNF**	**Curcumin**	**DMF**	**Control**
**Liver**	956 (1.6, 1.4–1.7)*	815 (1.4, 1.2–1.5)*	739 (1.2, 1.1–1.4)*	679 (1.1, 1.03–1.2)*	602
**Kidney**	92.5 (1.2, 1.0–1.3)	80 (1.0, 0.77–1.3)	65 (0.81, 0.67–1.0)	89 (1.1, 0.96–1.3)	81
**Prostate**	6.7 (1.2, 1.1–1.5)*	5.7 (1.06, 1.0–1.1)	6.9 (1.3,1.2–1.4)*	7.0 (1.3, 1.0–1.7)	5.4
**Bladder**	0.78 (4.2, 3.6–4.9)*	0.26 (1.4, 0.81–2.3)	0.28 (1.5, 0.96–2.3)	0.29 (1.5, 0.99–2.4)	0.19

Data are mean values of 8–10 animals in each group

* = P < 0.05 based on confidence intervals of the ratio of treated vs. control total GST specific activities

**Table 4:** Specific activity of GSTmu in nmol/min/mg of protein (Ratio of the treated/control enzyme specific activities, 95% Confidence Intervals)

	**Sulforaphane**	**BNF**	**Curcumin**	**DMF**	**Control**
**Liver**	0.09 (0.39, 0.25–0.62)*	0.20 (0.87, 0.71–1.1)	0.23 (1.0, 0.79–1.3)	0.29 (1.3, 1.0–1.6)	0.23
**Kidney**	0.06 (3.0, 0.92–9.7)	0.03 (1.5, 0.46–4.9)	0.21 (10, 3.2–34)*	0.02 (1.0, 0.25–4.0)	0.02
**Prostate**	0.73 (1.9, 1.4–2.4)*	0.76 (1.9, 1.5–2.5)*	0.67 (1.7,1.4–2.1)*	0.34 (0.87, 0.69–1.1)	0.39

Data are mean values of 8–10 animals in each group

* = P < 0.05 based on confidence intervals of the ratio of treated vs. control GST mu specific activities
